# Case Report: A hybrid approach of selective adrenal artery and venous embolization for the treatment of unilateral aldosterone-producing adenoma

**DOI:** 10.3389/fcvm.2025.1628754

**Published:** 2025-09-02

**Authors:** Changqiang Yang, Sen Liu, Peijian Wang

**Affiliations:** ^1^ Department of Cardiology, Department of Clinical Medicine, The First Affiliated Hospital of Chengdu Medical College, Chengdu, Sichuan, China; ^2^ Key Laboratory of Aging and Vascular Homeostasis, Chengdu Medical College of Sichuan Province, Chengdu, Sichuan, China; ^3^ Department of Cardiology, Clinical Research Center for Geriatrics of Sichuan Province, Chengdu, Sichuan, China

**Keywords:** aldosterone-producing adenomas, adrenal artery embolization, adrenal venous embolization, adrenal arteriography, adrenal venography

## Abstract

Selective adrenal artery embolization (SAAE) has emerged as a promising minimally invasive alternative to traditional therapies for primary aldosteronism (PA). However, incomplete ablation of aldosterone-producing adenomas (APA) tissue remains a key limitation. To address this challenge, we propose a novel hybrid approach integrating SAAE with selective adrenal venous embolization (SAVE), designed to optimize the therapeutic efficacy of APA. We report a 37-year-old man with confirmed unilateral PA. Left adrenal APA was diagnosed through computerized tomography and adrenal venous sampling. The patient underwent combined selective adrenal artery and venous embolization (SAAE-SAVE) for left-sided APA. Trans-catheter embolization of the dominant left adrenal artery and primary draining vein was performed using 2 ml anhydrous ethanol. This SAAE-SAVE protocol successfully normalized serum hypokalemia levels and achieved sustained blood pressure control at 1-month follow-up. SAAE-SAVE represents a transformative therapeutic alternative for APA treatment, and further research is needed to validate its safety and efficacy.

## Introduction

**Figure 1:**
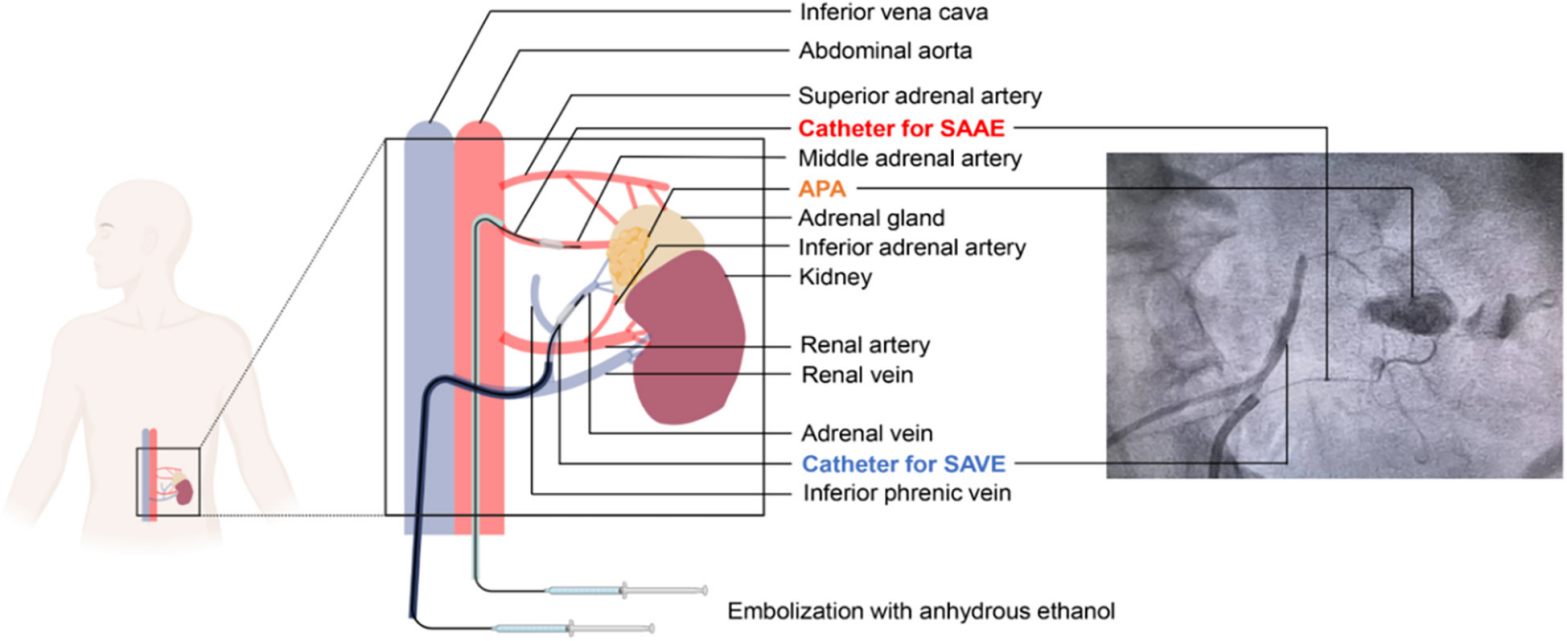
A hybrid approach of selective adrenal artery and venous embolization (SAAE-SAVE) for the treatment of aldosterone-producing adenomas. SAAE, superselective adrenal artery embolization; SAVE, superselective adrenal venous embolization.

Primary aldosteronism (PA), the most prevalent form of secondary hypertension, exhibits a severity-dependent prevalence ranging from 3.9% in stage 1 hypertension to 11.8% in stage 3 hypertension (1), with rates escalating to 17%–23% in resistant hypertension population (2). PA patients display an increased cardiovascular risk such as stroke, coronary artery disease, atrial fibrillation and heart failure, compared with patients with essential hypertension matched for age, sex and blood pressure (3). Etiologically, PA is broadly classified into six distinct subtypes. Unilateral disease, representing approximately 30% of cases, predominantly manifests as aldosterone-producing adenomas (APA) (4, 5). Current clinical guidelines recommend surgical adrenalectomy (laparoscopic or open approach) as the definitive therapy for unilateral PA (4, 6). Recent advances have expanded the therapeutic options to image-guided radiofrequency ablation techniques (CT-guided and endoscopic ultrasound-assisted), demonstrating promising APA-targeting efficacy (7–9). Notably, selective adrenal artery embolization (SAAE) has emerged as a minimally invasive alternative for PA and is under active investigation (10). However, the therapeutic efficacy in unilateral APA remains debated due to incomplete ablation of APA tissue, leading to residual hormonal activity and disease recurrence (11). Preclinical study in swine models demonstrated partial adrenal infarction via balloon-occluded retrograde ethanol injection into the adrenal vein (12). It was suggested that retrograde ethanol injection into the adrenal vein might provide a viable therapeutic strategy for APA (13). Building on this evidence, we proposed an innovative hybrid approach integrating SAAE with selective adrenal venous embolization (SAVE), designed to optimize the therapeutic efficacy (Figure 1). We reported a case of left-sided APA treated with the SAAE-SAVE combination approach and resolved the hypokalemia and hypertension.

## Case presentation

**Figure 2:**
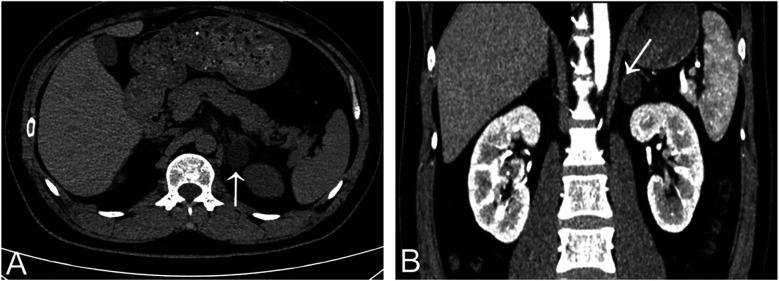
Adrenal gland CT scan showed a lipid-rich left adrenal nodule (arrows) [**(A)** non-contrast enhanced CT; **(B)** contrast-enhanced CT].

A 37-year-old man diagnosed with hypertension for 3 years was referred to the cardiology department for evaluation of possible secondary hypertension. He denied prior cerebrovascular accidents or cardiovascular diseases. Anthropometric measurements revealed a height of 175 cm and weight of 74 kg, yielding a body mass index (BMI) of 24.16 kg/m^2^. His peak recorded blood pressure (BP) was 185/130 mmHg. Despite daily therapy with nifedipine extended-release tablets 30 mg and sacubitril/valsartan 200 mg, BP control remained suboptimal: office BP was 160/110 mmHg, while 24 h ambulatory BP monitoring showed an average of 134/85 mmHg (daytime: 140/93 mmHg; nighttime: 124/73 mmHg). Laboratory findings were as follows (Table 1): hemoglobin 156 g/L, white blood cell count 10.07 × 10^9^/L, platelet count 243 × 10^9^/L; serum potassium 3.26 mmol/L, sodium 142.7 mmol/L, calcium 2.36 mmol/L; alanine aminotransferase (ALT) 60 U/L, aspartate aminotransferase (AST) 28 U/L; uric acid 455 μmol/L, triglycerides 1.96 mmol/L, total cholesterol 5.31 mmol/L, low-density lipoprotein cholesterol 3.73 mmol/L, and fasting blood glucose 4.95 mmol/L; creatinine 104.1 *μ*mol/L, estimated glomerular filtration rate (eGFR) 80.22 ml/min/1.73 m^2^. Routine urinalysis revealed no abnormalities (negative for protein, glucose, ketones, and leukocytes). To avoid interference with aldosterone-to-renin ratio (ARR) assays, antihypertensive therapy was switched to doxazosin 8 mg daily and verapamil 240 mg daily over a one-month period. Direct renin concentration and aldosterone levels were measured using a chemiluminescent immunoassay platform at our center. Hormonal evaluation in the upright position revealed: plasma aldosterone concentration (PAC) 937.18 pmol/L, suppressed renin 1.60 pg/ml, and ARR 585.74. In the supine position, results showed: PAC > 1,000 pmol/L, suppressed renin 1.71 pg/ml, and incalculable ARR due to unmeasurably high aldosterone levels. Circadian rhythm of serum cortisol and adrenocorticotropic hormone, plasma free metanephrines, and thyroid function were within normal range. The combination of markedly elevated aldosterone and suppressed renin suggested probable primary aldosteronism (PA). A supine saline suppression test was subsequently performed, involving intravenous infusion of 2 L of 0.9% sodium chloride solution over 4 h, with blood samples collected at baseline and post-infusion. Results demonstrated: baseline PAC > 1,000 pmol/L, renin 1.28 pg/ml; Post-infusion PAC > 1,000 pmol/L, renin 1.04 pg/ml. These findings confirmed autonomous aldosterone excess, supporting a PA diagnosis. Contrast-enhanced adrenal computed tomography (CT) scan identified a 1 cm left adrenal nodule with an attenuation value of 1 Hounsfield unit (HU), consistent with a lipid-rich adenoma (Figure 2).

**Table 1:** Preoperative and post-operative laboratory results.

Variables	Preoperative	POD 1	POD 2	POD 3	Postoperative 1 Month	Reference
Complete Blood Count						
Hemoglobin (g/L)	156	166	NA	NA	151	120–160
WBC (10^9^/L)	10.07	14.44	NA	NA	8.02	4–10
Platelet (10^9^/L)	243	231	NA	NA	265	100–300
Serum electrolytes						
Potassium (mmol/L)	3.26	2.45	2.99	3.03	4.2	3.5–5.3
Sodium (mmol/L)	142.7	141.7	142.7	139.6	138.2	137–147
Calcium (mmol/L)	2.36	2.28	2.21	2.24	2.34	2.11–2.52
Liver function						
ALT (U/L)	60	52	NA	NA	48	9–50
AST (U/L)	28	32	NA	NA	30	15–40
Metabolic panel results						
UA (*μ*mol/L)	455	557	NA	NA	420	200–415
TG (mmol/L)	1.96	NA	NA	NA	1.87	<2.3
TC (mmol/L)	5.31	NA	NA	NA	5.01	<5.17
LDL-C (mmol/L)	3.73	NA	NA	NA	2.51	<3.36
HDL-C (mmol/L)	1.30	NA	NA	NA	1.12	> 1.04
FBG (mmol/L)	4.95	9.49	NA	NA	5.32	3.89–6.11
Renal function						
Creatinine (μmol/L)	104.1	94.6	NA	NA	88.9	17.7–107
eGFR (ml/min/1.73m2)	80.22	90.06	NA	NA	97.09	
Hormonal profile						
Supine Renin (pg/ml)	1.71	1.14	1.14	3.74	NA	2.4–32.8
Supine Aldosterone (pg/ml)	>1,000	175.29	80.61	68.00	NA	10–160
Supine ARR	NA	93.97	70.71	18.18	NA	
Upright Renin (pg/ml)	1.60	NA	NA	NA	4.52	3.8–38.8
Upright Aldosterone (pg/ml)	937.18	NA	NA	NA	78.25	40–310
Upright ARR	585.74	NA	NA	NA	17.31	
Clinical data						
SBP (mmHg)	140–150	140–160	135–150	110–135	132	
DBP (mmHg)	78–110	80–105	73–100	70–95	82	
HR (bpm)	65–80	83–95	80–90	77–85	75	
Antihypertensive drugs	Doxazosin 8 mg and Verapamil 240 mg	Without Drugs	Without Drugs	Without Drugs	Without Drugs	

POD, postoperative day; WBC, white blood count; ALT, alanine aminotransferase; AST, aspartate aminotransferase; UA, uric acid; TG, triglycerides; TC, total cholesterol; LDL-C, low-density lipoprotein cholesterol; HDL-C, high-density lipoprotein cholesterol; FBG, fasting blood glucose; eGFR, estimated glomerular filtration rate; ARR, aldosterone renin ratio; SBP, systolic blood pressure; DBP, diastolic blood pressure; HR, heart rate.

Adrenal venous sampling (AVS) without adrenocorticotropic hormone stimulation was performed via the femoral vein approach. Blood samples were collected selectively from the right adrenal vein (RAV), left adrenal vein (LAV), and the distal inferior vena cava (IVC). AVS results were presented in Table 2. The lateralization index (LI) for the left adrenal gland was 44.1, with a contralateral suppression index (CSI) of 0.036 for the right adrenal gland. These findings indicated excessive aldosterone secretion from the left adrenal gland, consistent with the international consensus for subtyping PA (14). Surgical resection of the left adrenal gland was recommended; however, the patient declined surgery and expressed unwillingness to undergo long-term mineralocorticoid receptor antagonist therapy. Following obtainment of written informed consent, a novel hybrid approach termed SAAE-SAVE using anhydrous ethanol was performed.

**Table 2:** Adrenal vein sampling results.

Sample	A (pg/ml)	C (nmol/L)	A/C	SI	LI	CSI
IVC	1,422.00	273.2	5.20	NA	NA	NA
RAV	1,630.00	8,381.0	0.19	30.67	NA	0.036
LAV	47,825.00	5,706.1	8.38	20.88	44.10	NA

A, aldosterone; C, cortisol; A/C, aldosterone-to-cortisol ratio; SI, selective index; LI, lateralization index; CSI, contralateral suppression index; IVC, inferior vena cava; RAV, right adrenal vein; LAV, left adrenal vein.

**Figure 3:**
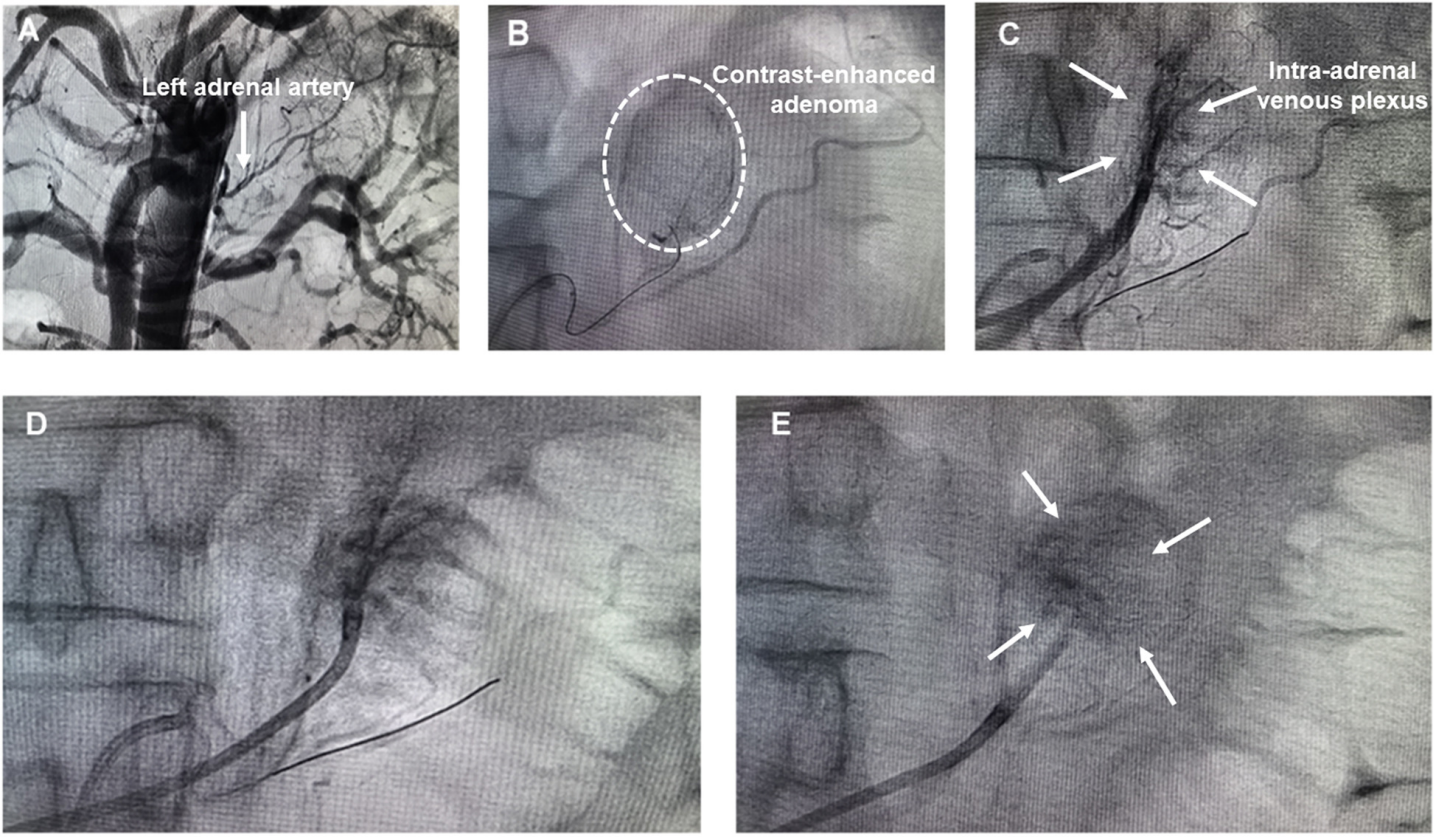
**(A)** Abdominal aortography; **(B)** left adrenal arteriography; **(C)** simultaneous left adrenal venography and arteriography; **(D)** left adrenal arteriography post embolization; **(E)** left adrenal venography post embolization.

Preoperatively, the patient received antihypertensive therapy with doxazosin (8 mg once daily) and verapamil (240 mg once daily). His blood pressure was maintained at 140–150/78–110 mm Hg with a heart rate of 65–80 beats/min (Table 1). The SAAE procedure was performed through right femoral arterial and venous access under local infiltration anesthesia, as previously reported (15). A 6-Fr Judkins right (JR 3.5) catheter (Launcher, Medtronic, USA) was inserted into the femoral artery through a 6-Fr sheath and the catheter was positioned to abdominal aorta at the level of vertebrae T 12-L 1. Non-selective arteriography was performed to visualize the left adrenal arteries (Figure 3A). A 6-Fr Judkins (JR 3.5) guiding catheter was employed for super-selective cannulation of the left adrenal artery, followed by the insertion of a Fielder XT-R guidewire. After positioning the micro-catheter within the proximal to mid segments of the left adrenal artery, adrenal arteriography was performed to identify the tumor-supplying artery of the aldosterone-producing adenoma (APA) and delineate the entire adenoma staining area (Figure 3B). Concurrently, a second catheter was introduced via femoral vein access and advanced distally into the left adrenal central vein draining functional adenomas, with careful avoidance of the inferior phrenic venous branch. Prudent retrograde venography and adrenal arteriography were performed concurrently to delineate the anatomy of the adrenal venous plexus and the adenoma (Figure 3C). To minimize the risk of anhydrous ethanol backflow entering non-target arteries, the balloon dilation catheter was inflated using a dedicated inflation device prior to anhydrous ethanol injection. For prophylactic analgesia, a 5 mg of morphine was administered intravenously during the procedure. The targeted left adrenal artery was then embolized under fluoroscopic guidance via slow, controlled injection of 2.0 ml of lidocaine followed by 2.0 ml of anhydrous ethanol through a micro-catheter. Post-embolization angiography demonstrated absence of ante-grade flow within the feeding arteries and attenuation of tumor staining, confirming the technical success of the procedure (Figure 3D). To ensure comprehensive embolization of the APA, the catheter was repositioned to the intra-adrenal venous plexus, and balloon-occluded retrograde trans-venous ethanol injection was performed. Specifically, a micro-balloon catheter was positioned in the left adrenal vein. Subsequently, anhydrous ethanol was administered retrogradely under balloon occlusion to induce partial infarction within the APA. Post-procedural venography confirmed markedly reduced opacification of the intra-adrenal venous plexus, indicating successful embolization (Figure 3E). During the SAAE and SAVE procedure, both intra-arterial and intravenous injections of anhydrous ethanol induced acute BP elevation, with blood pressure rapidly escalating to 200/120 mmHg. Sodium nitroprusside infusion was immediately initiated under continuous arterial pressure monitoring. This intervention effectively prevented further blood pressure elevation and achieved a controlled reduction to baseline levels within 3–5 min. Consequently, transient hypertension throughout the entire surgical procedure was successfully managed by intravenous infusion of sodium nitroprusside.

The patient was transferred to the coronary care unit postoperatively and received continuous electrocardiographic monitoring until postoperative day 3. Post-intervention hematological parameters and clinical data were summarized in Table 1. Post-intervention assessments demonstrated a gradual normalization of the aldosterone-to-renin ratio (ARR), and blood pressure progressively declined and ultimately stabilized within the normal range. On postoperative day 1 (POD1), a significant decrease in aldosterone levels (PAC = 175.29 pg/ml) was noted. However, renin suppression persisted (renin = 1.14 pg/ml) and ARR still elevated (ARR = 93.97). By POD2, aldosterone concentrations further declined to near-physiological ranges (PAC = 80.61 pg/ml), while renin-aldosterone axis dysfunction persisted (renin = 1.14 pg/ml, ARR = 70.71). Notably, physiological equilibrium was restored by POD3, characterized by complete resolution of renin suppression (renin = 3.74 pg/ml), sustained decrease in aldosterone concentrations (PAC = 68.00 pg/ml) and ARR normalization (ARR = 18.18), indicating restored negative feedback mechanisms. The patient was discharged on POD 3**.** At the 1-month follow-up, serum potassium concentration remained within normal range (4.2 mmol/L; reference: 3.5–5.0 mmol/L) without potassium supplementation. Both clinic blood pressure (132/82 mmHg) and 24 h average blood pressure (124/71 mmHg) were maintained within acceptable limits off antihypertensive therapy. Concurrent aldosterone-renin ratio testing revealed persistently low serum aldosterone (PAC = 78.25 pg/ml) alongside complete reversal of prior renin suppression (renin = 4.52 pg/ml). These results indicated that the patient achieved complete biochemical success and clinical cure of hypertension during the observation period, according to the PASO criteria (16).

## Discussion

We reported a case of treatment-refractory hypertension and persistent hypokalemia secondary to unilateral PA caused by a left adrenal aldosterone-producing adenoma. The patient was successfully managed using a novel hybrid approach combining selective adrenal artery and venous embolization (SAAE-SAVE) with anhydrous ethanol, achieving complete biochemical remission and hypertension resolution.

It is 70 years since the discovery of PA (17). For much of this period, management of unilateral PA has scarcely changed since the advent of laparoscopic adrenalectomy in the 1990s. The imperative for clinical innovation is driven by overwhelming evidence that PA represents the most common potentially curable etiology of hypertension (1). SAAE has emerged as an alternative therapeutic option for PA patients who are either unwilling or unable to undergo surgical adrenalectomy and cannot tolerate or refuse mineralocorticoid receptor antagonists (15). Compared to traditional surgical approaches, SAAE is less invasive and associated with a lower incidence of complications. However, the therapeutic efficacy of SAAE remains subject to debate, especially in unilateral PA (18). Previous article published by Hokotate et al. evaluated the effectiveness and long-term follow-up results of SAAE in 33 aldosteronoma cases (11). SAAE was successful in 27 (82%) of 33 patients. In one patient, aldosteronoma recurred 15 months after SAAE. In addition, a recent article published by Sun et al. compared the clinical and biochemical outcomes of adrenalectomy (*n* = 52) with catheter-based adrenal ablation (trans-arterial embolization) (*n* = 60) for unilateral aldosterone-producing adenoma patients at 6 months of treatment (19). According to the PASO criteria (16), complete clinical success was achieved in 21 (40.4%) patients in the ablation group vs. 33 (55.0%) patients in the adrenalectomy group. Complete biochemical success was achieved in 30 (57.7%) patients in the ablation group vs. 51 (85.0%) patients in the adrenalectomy group. The efficacy of SAAE for unilateral APA in the present study was lower than those undergoing surgical adrenalectomy. A potential explanation is that SAAE may not achieve complete destruction of the entire APA, resulting in only partial destruction of the adenoma confirmed by ^68^Ga-Pentixafor PET/CT images (20). Previous study has demonstrated that adrenal ablation by retrograde venous ethanol injection is available (21). Therefore, we firstly introduced the selective adrenal artery and venous embolization (SAAE—SAVE) technique for the management of adrenal aldosterone-producing adenomas.

In this case, selective adrenal artery angiography identified the feeding arterial branches of the APA. Trans-catheter intra-arterial injection of anhydrous ethanol into the APA's blood supply allowed diffusion into the adenoma tissue via bloodstream, inducing targeted necrosis. Concurrently, ethanol-mediated vascular occlusion interrupted perfusion, further promoting lesion devitalization. In this case, post-embolization angiography confirmed disappearance of tumor staining and adrenal arterial occlusion. This dual mechanism achieved the therapeutic goal of normalizing aldosterone secretion. Given incomplete ablation of APA tissue, we performed retrograde administration of anhydrous ethanol via the adrenal venous system to achieve chemical ablation. Pre-procedural retrograde venography precisely delineated adrenal venous plexus anatomy. Following micro-balloon catheter insertion into the left adrenal vein, balloon occlusion during ethanol infusion induced targeted partial APA infarction. Post-procedural venography demonstrated significantly reduced intra-adrenal plexus filling. This approach achieved: 1. marked aldosterone reduction, 2. complete biochemical cure of PA, and 3. sustained hypertension resolution throughout follow-up.

As with all novel therapeutic interventions, promising efficacy and safety warrant careful evaluation to mitigate premature implementation. In the case report, during the SAAE and SAVE procedures, both intra-arterial and intravenous injections of anhydrous ethanol induced acute BP elevation. The BP rapidly escalated to 200/120 mmHg, a phenomenon primarily attributed to catecholamine release triggered by ethanol-induced ablation of adrenal cells. This mechanism was corroborated by our previous study demonstrating a 3- to 100-fold increase in catecholamine levels (including dopamine, epinephrine, and norepinephrine) via ipsilateral adrenal vein sampling during SAAE (15). These findings suggest that ethanol injection may cause destruction of adrenal medullary tissue, resulting in rapid release of substantial catecholamine stores, thereby contributing to the observed hemodynamic instability. Although, it seems a dangerous procedure, sodium nitroprusside infusion under continuous arterial pressure monitoring can effectively prevent further BP elevation and achieved controlled reduction to baseline levels within 3–5 min. Consequently, transient hypertension throughout the combined SAAE and SAVE procedures can be effectively managed via sodium nitroprusside administration. In addition, the current SAAE-SAVE technique using anhydrous ethanol is anatomically restricted to left-sided aldosterone-producing adenomas (APAs) due to consistent left adrenal vein anatomy (22). Technical application to right-sided APAs remains challenging given the right adrenal vein's characteristically short length, which complicates catheter placement (23). Future prospective studies must validate SAAE-SAVE's safety and efficacy profile while establishing formal patient selection criteria. Long-term monitoring of adrenal function and cardiovascular outcomes will ultimately determine its viability as a potential alternative to laparoscopic adrenalectomy.

## Conclusions

SAAE-SAVE represents a transformative therapeutic alternative for aldosterone-producing adenoma. Further broader validation of its safety-efficacy profile across diverse clinical scenarios remains is warranted.

## Data Availability

The raw data supporting the conclusions of this article will be made available by the authors, without undue reservation.
